# An Overview of Heterogeneous Catalysts Based on Hypercrosslinked Polystyrene for the Synthesis and Transformation of Platform Chemicals Derived from Biomass

**DOI:** 10.3390/molecules28248126

**Published:** 2023-12-15

**Authors:** Oleg Manaenkov, Linda Nikoshvili, Alexey Bykov, Olga Kislitsa, Maxim Grigoriev, Mikhail Sulman, Valentina Matveeva, Lioubov Kiwi-Minsker

**Affiliations:** 1 Department of Biotechnology, Chemistry and Standardization, Tver State Technical University, 170026 Tver, Russia; ovman@yandex.ru (O.M.); nlinda@yandex.ru (L.N.); bykovav@yandex.ru (A.B.); kislitza@yandex.ru (O.K.); ge.max2015@yandex.ru (M.G.); science@science.tver.ru (M.S.); valen-matveeva@yandex.ru (V.M.); 2 Ecole Polytechnique Fédérale de Lausanne, ISIC-FSB-EPFL, CH-1015 Lausanne, Switzerland

**Keywords:** porous organic polymer, hypercrosslinked polystyrene, platform chemicals, heterogeneous catalysts, metal nanoparticles, biomass processing

## Abstract

Platform chemicals, also known as chemical building blocks, are substances that serve as starting materials for the synthesis of various value-added products, which find a wide range of applications. These chemicals are the key ingredients for many fine and specialty chemicals. Most of the transformations of platform chemicals are catalytic processes, which should meet the requirements of sustainable chemistry: to be not toxic for humans, to be safe for the environment, and to allow multiple reuses of catalytic materials. This paper presents an overview of a new class of heterogeneous catalysts based on nanoparticles of catalytically active metals stabilized by a polymer matrix of hypercrosslinked polystyrene (HPS). This polymeric support is characterized by hierarchical porosity (including meso- and macropores along with micropores), which is important both for the formation of metal nanoparticles and for efficient mass transfer of reactants. The influence of key parameters such as the morphology of nanoparticles (bimetallic versus monometallic) and the presence of functional groups in the polymer matrix on the catalytic properties is considered. Emphasis is placed on the use of this class of heterogeneous catalysts for the conversion of plant polysaccharides into polyols (sorbitol, mannitol, and glycols), hydrogenation of levulinic acid, furfural, oxidation of disaccharides, and some other reactions that might be useful for large-scale industrial processes that aim to be sustainable. Some challenges related to the use of HPS-based catalysts are addressed and multiple perspectives are discussed.

## 1. Introduction

In recent decades, the scientific world has developed a clear understanding of the future of the development of the chemical and fuel industries, in which an important place will be occupied by platform chemicals synthesized from biomass [1], primarily from lignocellulose produced from forestry and agriculture waste, the volume of which reaches 200 billion tons per year [2]. In 2004 the US Department of Energy compiled a list of biomass-derived chemicals with the highest added value. These include the following: 1,4-diacids (succinic, fumaric, malic acids), 2,5-furandicarboxylic acid, 3-hydroxypropionic acid, aspartic acid, glucaric acid, glutamic acid, itaconic acid, levulinic acid, 3-hydroxybutyrolactone, and glycerol, as well as sugar alcohols (sorbitol, xylitol, etc.) [3]. This list was updated in 2010 [4], when ethanol, furfural, 5-hydroxymethylfurfural, isoprene, and lactic acid were added. All these substances underlie many sequences of chemical transformations aimed at the synthesis of polymers [5], biofuels [6], and raw materials for the chemical industry [7]. The volume of platform chemicals derived from biomass is constantly growing and today already represents hundreds of billions of US dollars annually [8], which in mass terms approaches approximately 100 million tons per year [9,10]. Various large chemical companies, such as DuPont, BASF AG, Dow Chemical, and many others are developing technologies for the conversion of biomass into platform chemicals and their integration into existing processes [8]. The current energy crisis is also a driving force for the market growth of commercially valuable biomass-derived chemicals and is expected to increase by 16% in 2025 [11], which in monetary terms should reach 867.7 billion US dollars [12]. The most attractive biomass is lignocellulosic, since it does not provide any nutritional value [13].

To ensure the sustainable processing of biomass, it is necessary to address fundamental economic, social, and environmental requirements. For example, none of the strategies for the pretreatment of lignocellulosic biomass have proven to be environmentally friendly and safe [14]. The strong structure of microcrystalline cellulose makes it difficult to involve plant biomass directly in conversion processes. The dissolution and destruction of cellulose macromolecules requires the use of complex and environmentally harmful solvents (ionic liquids, mineral acids, etc.), expensive and non-regenerable enzymes, and/or “harsh” reaction conditions (high temperatures and pressures) [15].

One effective solution is heterogeneous catalysts designed for a required range of reaction conditions [16]. However, the use of catalysts also brings a number of problems related to their stability under hydrothermal conditions, toxicity, high selectivity to the target products, etc. [17]. In this regard, an urgent task is to develop new catalytic systems that are stable under “harsh” conditions of plant biomass conversion, have high activity and yields of target products, and are safe for humans and the environment. Hypercrosslinked polymers are very promising materials, which present many properties of effective heterogeneous catalysts, like developed specific surface area (from hundreds to few thousands of m^2^/g), controlled porosity, possibility of surface functionalization, exceptional adsorption properties [18,19], chemical and thermal stability, and low cost [20]. In addition, some hypercrosslinked polymers can be synthesized from monomers of biological origin or related compounds [21], for example using lignin, catechol, and starch, which in combination with a mechanochemical method (without any use of toxic chlorine-containing solvents) fully complies with the principles of green chemistry [22,23]. The carbonizates of hypercrosslinked polymers also look promising for use as catalysts [24]. However, despite the advantages, the use of hypercrosslinked polymers as heterogeneous catalysts is not always straightforward. The main disadvantages of catalysts based on hypercrosslinked polymers can be summarized as follows: low mechanical strength, leaching of active metals, sensitivity to oxidation, restricted temperature (<300–400 °C), slow diffusion in micropores, and difficult access of large molecules to the active centers.

In general, it should be noted that hypercrosslinked polymers are already widely applied in industry as excellent (ad)sorbents. Their use for the synthesis of heterogeneous catalysts is a relatively new but dynamically developing area. This article provides an overview of a new class of heterogeneous catalysts based on nanoparticles of catalytically active metals stabilized in a polymer matrix of hypercrosslinked polystyrene (HPS), their synthesis, properties, and application to produce several platform chemicals from renewable raw materials. Some transformations of platform chemicals into compounds with high added value are addressed and multiple perspectives are discussed.

## 2. Hypercrosslinked Polystyrene (HPS): Synthesis, Properties, and Applications

The year 2019 marked half a century since the synthesis of the first HPS samples, which are known as the third generation of polystyrene networks, or “Davankov-type resins” [25,26,27]. According to the synthesis method proposed by V.A. Davankov, polystyrene chains are crosslinked in solution with rigid bridges (crosslinking agents)—bis-chloromethyl derivatives of aromatic hydrocarbons or monochlorodimethyl ether. The latter reacts with polystyrene in the presence of a Friedel–Crafts catalyst in two stages: (1) introduction of chloromethyl groups into polystyrene and (2) binding of phenyl rings by a methyl group. As a result, a rigid and relatively long bridge is formed, containing a fragment of the crosslinking agent and two phenyl rings of the original polystyrene [26]. Over the decades, the synthesis process has been improved and modified. For example, a one-pot preparation of HPS sorbents was recently proposed, which is based on the in situ formation of monochlorodimethyl ether (MCDE) through the reaction of methylal with acid chlorides of organic and inorganic acids, in particular, CH_3_COCl, (COCl)_2_, SO_2_Cl_2_, and POCl_3_ [28]. The application scope of HPS has now expanded enormously. Methods and strategies for their synthesis have been developed in order to control the microporosity/mesoporosity ratio, to introduce functional groups into the polymer, to improve the selective sorption of gases, organic aqueous pollutants, etc. This new class of microporous materials presents high-performance characteristics [29]. Due its multiple advantages, such as a variety of synthesis methods, ease of functionalization, large specific surface area (SSA), use of inexpensive reagents [30], mild synthesis conditions, and chemical and thermal stability, HPS is increasingly attracting research interests [31]. HPS has become a universal platform for the synthesis of nanoporous materials with specified properties, which, in addition to traditional application in chromatography, gas adsorption, and sorbents for water treatment, have begun to be used in drug delivery systems, sensors, and heterogeneous catalysts [27].

**Figure 1:**
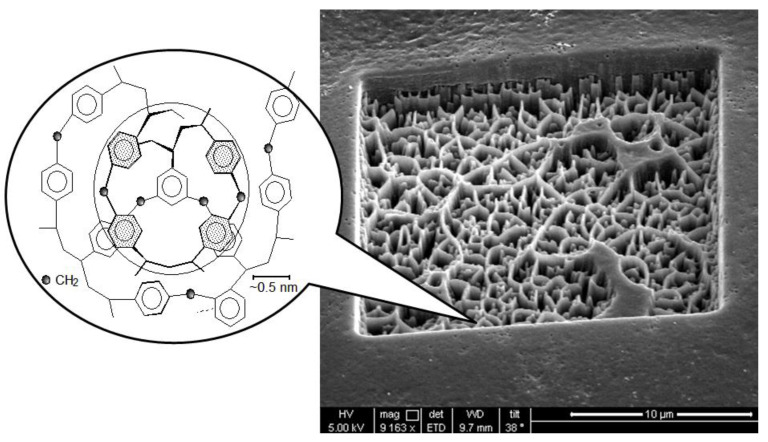
Schematic presentation of porous structure of hypercrosslinked polystyrene (HPS) and its scanning ion conductance microscopy image.

Hypercrosslinked polystyrene is a very rigid polymer network with a special type of porosity (Figure 1). The pore size is quite small (about 20 Å) and, according to the IUPAC nomenclature, is on the border of micro- and mesoporosity [32]. The nature of porosity and specific surface area of HPS strongly depend on the characteristics of the initial reagents [33], the method of polymer synthesis and is about 600–2000 m^2^/g or even more [34,35,36,37]. The exceptional sorption properties of HPS are based mainly on hydrophobic and π–π interactions [38], but through functionalization the possibilities of using HPS as a sorbent are significantly expanded [39]. Its chemical modification can be carried out after polymer synthesis, for example, via treatment with concentrated sulfuric acid to sulfonate the surface [25,40] or by introducing monomers containing functional groups directly during the synthesis of HPS [41,42,43].

Due to its properties, HPS has gained great commercial popularity over the past decades. Many companies produce it for large-scale sorption processes in the chemical, food, and water treatment industries, for example, Purolite (Hypersol-Macronet, MN series), Dow Chemical (Optipore), Lanxess (Lewatit VP OC 1163 and S 7768), and Jiangsu N&G Environmental Technology (NG-99 and NG-100), and for analytical purposes, such as International Sorbent Technology (Isolute ENV+), Merck (LiChrolut EN), Spark Holland (HySphere), Macherey Nagel (HR-P), etc. [26,27]. The range of manufactured HPS includes neutral sorbents with various porous structure, strong and weakly basic anion exchangers and cation exchangers, sorbents for high-performance liquid chromatography (HPLC) and cartridge fillers for solid-phase extraction. Their combined abilities of quickly absorbing significant amounts of different gases and thermodynamically compatible and incompatible solvents with their capacity for rapid regeneration makes hypercrosslinked polystyrenes promising adsorption materials, including for their uses in liquid chromatography and solid-phase extraction [44].

There is a significant amount of research on the application of HPS-based sorbents in medicine. Thus, Tolmacheva et al. [45] used HPS Diapak P-3 (Biokhimak, Russia) for the adsorption of catecholamines (adrenaline, norepinephrine, and dopamine), which can serve as biomarker of heart disease, diabetes, schizophrenia, etc. The average recovery of catecholamines from HPS was 89–98%, showing a promise for isolation and concentration of catecholamines from aqueous media (including biological fluids). Hemosorption is an actively developing method of removing toxic substances from humans by pumping the blood stream through a cartridge filled with a sorbent, including one based on HPS [46]. This procedure is used not only for emergencies (poisoning and severe infections), but also for the treatment of chronic diseases (asthma, allergies, pancreatitis, etc.) [26,47,48]. For example, in [49] a new adsorbent based on hypercrosslinked polystyrene modified with pyridinyl—HCP (St-DVB-VP)—was used to remove bilirubin and medium/large-molecular toxins (IL-6 and PTH) from a liver. In addition to good adsorption properties towards toxins, this sorbent showed a low rate of hemolysis, a low risk of blood clotting, and acceptable hemocompatibility. Pautova et al. [50] isolated nine aromatic microbial metabolites from blood, using the microextraction by packed sorbent (MEPS) method in combination with an HPS-based sorbent. Based on the results obtained, the authors concluded that the use of HPS in commercial MEPS devices is promising for the analysis of biological samples for early diagnosis of sepsis and for monitoring the effectiveness of treatment. The use of HPS for preconcentration of analytes made it possible to reduce the detection limit of antibiotics of the tetracycline group (tetracycline, oxytetracycline, chlortetracycline, and doxycycline) by 90–100 times (0.6–2.0 ng/mL) [51]. Tolmacheva et al. assessed the adsorption properties of a magnetic adsorbent based on hypercrosslinked polystyrene (HCPS-Fe_3_O_4_) towards tetracycline antibiotics [52]. The authors showed that HCPS-Fe_3_O_4_ retains adsorption properties towards tetracyclines and is easily separated from the solution by applying a magnetic field.

It should be noted that materials with magnetic properties, including sorbents and catalysts, increasingly find applications in various fields of research and industry [53,54,55,56,57]. From this point of view, magnetic HPS also deserve attention, since they combine the technological advantages of magnetic materials and HPS’ high adsorption capacities [44,58]. There are several options for the preparation of magnetic HPS: (1) synthesis of a polymer network in the presence of magnetite nanoparticles (Fe_3_O_4_) [59,60]; (2) synthesis of magnetic nanoparticles directly in the polymer matrix, for example, via the precipitation of magnetite [61]; and (3) sorption of presynthesized magnetite nanoparticles on the HPS surface [44]. Thus, Pastukhov [62] synthesized magnetic composites using the industrial polymer adsorbents Macronet MN270, 200, 202, and 600 as a basis; these belong to the class of hypercrosslinked polystyrenes, styrene-divinylbenzene sorbent Amberlite XAD4 and a hypercrosslinked copolymer of styrene and divinylbenzene. Magnetic sorbents were obtained by immobilizing magnetite nanocrystallites in the pores of HPS via chemical deposition. It has been shown that synthesized composites based on industrial biporous HPS are capable of adsorbing well organic compounds of various classes: aliphatic and aromatic hydrocarbons, alcohols, and ethers. The sorption value of particularly toxic organic compounds—dioxane, benzene, and carbon tetrachloride—reaches 0.8 mL/cm^3^. Furthermore, based on the industrial sorbent Macronet MN270, but using a different technique based on the thermal destruction of iron salts, a magnetic composite Fe_3_O_4_/HPS MN270 was synthesized. It was characterized by a high saturation magnetization value (4.5 emu/g) and a magnetite particle size of 40 ± 5 nm [63], which was used for the synthesis of magnetic heterogeneous catalysts. A convenient method has been proposed for producing a Fe-containing HPS composite based on a waste foam plastic [64]. This is a fast method that uses readily available reagents and solves the problem of disposing of bulky foam waste. The resulting product can be used for the sorption of hydrogen sulfide and toxic organic solvents, as well as the decomposition products of natural residues—putrescine, cadaverine, indole, and skatole.

Over the last decades, science and industry have accumulated significant knowledge in the use of HPS as a sorbent for chromatography and technological processes for the purification of aqueous and organic media. However, new materials appear constantly. Thus, Gui et al. [65] reported the synthesis of hypercrosslinked polystyrenes (sPSs)–HCL-X prepared from syndiotactic polystyrene (sPS) aerogels via Friedel–Crafts alkylation. sPSs have a hierarchical porous structure on three scales: macropores connecting to macropores, mesopores, and micropores. HCL-X possesses SSA up to 931 m^2^/g and the ability to instantly absorb large amounts of organic liquids (up to 61 mL/g). The authors presented HCL-X as a good candidate for removing oils from oil–water mixtures and for producing carbonates with hierarchical pores. The functionalization of HPS opens up possibilities for the synthesis of selective sorbents. For example, Liao et al. [66] reported the synthesis of hypercrosslinked microporous poly(para-methoxystyrene) (HCPMOS) functionalized with methoxy groups, capable of selectively extracting Fe^3+^ from aqueous solutions even at a very low concentration of 10 ppm. The authors attribute the high selectivity of HCPMOS towards iron cations to their stronger interactions with methoxy groups as compared to cations of other metals. A number of methylamine-modified hypercrosslinked resins have been proposed for the selective sorption of citric acid [67]. During the periodic sorption of citric acid, the methylamine-modified HM-65-2 resin showed a high adsorption capacity (136.3) mg/g and a selectivity of 6.98 (citric acid/glucose), which makes this sorbent a promising candidate for the purification of citric acid and further use in the separation process with simulating a moving bed (SMB). This method is used to separate one or more chemical compounds from one or more other chemicals and allows the production of large quantities of highly purified material at a reduced cost. In the SMB technique instead of moving the bed, the feed inlet, the solvent or eluent inlet, and product exit positions are moved continuously, giving the impression of a moving bed, with continuous flow of solid particles and continuous flow of liquid in the opposite direction of the solid particles. SMB is increasingly applied as a separation technique in the pharmaceutical industry, production of fine chemicals, and in the field of bioengineering [68].

A very promising area of practical application of HPS is the adsorption and storage of gases, primarily carbon dioxide [69]. For example, hypercrosslinked polystyrene microspheres with a specific surface area of 1161 m^2^/g and a total pore volume of 0.72 cm^3^/g are capable of reversibly storing up to 2.27 wt.% H_2_ at 1 bar/77.3 K and 14.8 wt.% CO_2_ at 1 bar/273 K [70], which makes them a promising packing material for HPLC, adsorbents for organic compounds, and materials for gas storage. Pan et al. [71] developed an inexpensive and efficient method for the synthesis of amino-functionalized hypercrosslinked polymer nanoparticles (AHCPNPs) with well-defined spherical morphology, high specific surface area (507.64 m^2^/g) and excellent CO_2_ adsorption capacity of 53.65 wt.% (12.21 mmol/g). The authors showed the possibility of regulating the adsorption properties of AHCPNPs by selecting diblock copolymers with different molecular weights or adjusting the ratio of the original diblock copolymers to polystyrene. An interesting fact is that in some cases, a decrease in SSA after modification of a hypercrosslinked polymer does not lead to a decrease in its adsorption properties. Moradi et al. [72] modified the surface of HPS with amino groups, and the SSA decreased almost twofold—from 806 m^2^/g to 453 m^2^/g—but the ability of the modified polymer to absorb CO_2_, on the contrary, increased from 301.67 to 414.41 mg/g. In spite of a slight decrease in the sorption capacities of these adsorbents after recycling, the authors believe that the developed materials are suitable for industry.

## 3. Hypercrosslinked Polystyrene in Heterogeneous Catalysis

One of the promising areas for practical use of porous organic polymers, including hypercrosslinked polystyrenes, is heterogeneous catalytic systems [73]. The large pore volume, large specific surface area, wide possibilities for functionalization, and the ability to control the particle size of the active phase are responsible for the rapid growth in the number of reports on the synthesis of polymer-based catalysts, as evidenced by recent publications [74,75,76,77]. Different catalytic systems based on porous polymers have been reported, like sulfonated solid acid catalysts for hydrolysis, dehydration, and esterification processes [78,79,80,81], systems for photocatalysis [82,83], hydrogenation [84], and many others. Despite the multiple problems (structural damage, leaching of active metals, swelling, difficult access of large substrate molecules to active centers in micropores, etc.), it is expected that hypercrosslinked polymers will make a significant contribution to the development of novel heterogeneous catalysts in the coming years [85].

The first reports on the metal nanoparticles stabilized in the microporous structure of HPS were made by Sidorov et al. [86]. Impregnation of HPS with solutions of cobalt compounds followed by thermolysis at 200 °C led to the formation of discrete spherical Co nanoparticles with sizes in the range of 1–3 nm. The authors have shown that particle size is controlled by nano sized HPS cavities, which physically limit the increase in their size. One of the first examples of polymer-based hypercrosslinked polystyrene catalysts was a Pt-containing HPS [87]. The microporous structure of HPS made it possible to obtain Pt-nanoparticles of 1.3 nm. The catalyst was used in the oxidation of L-sorbose to 2-keto-L-gulonic acid, demonstrating selectivity for the target product of 98% at 100% conversion. Further studies on the synthesis of catalysts based on commercial HPS, namely micro/macroporous Macronet NM (Purolite, Llantrisant, UK), and different noble metals, showed that the catalytic activity of such systems depends, in particular, on the presence of macropores [88], chemical nature of the precursor of the active phase [89], the presence of functional groups in the HPS [90], and the type of solvent used for the reaction [91]. In general, such catalytic systems have shown higher efficiency as compared to the catalysts with traditional catalytic supports [29].

Further research on HPS-based catalysts is aimed on the synthesis with different active phase, HPS of different porosities and with functional groups, as well as on expanding the scope of reactions. Thus, 1% Pd on HPS was used in the hydrodeoxygenation of stearic acid giving a heptadecane yield of up to 97% [92]. A new method for the synthesis of Pd-containing catalysts based on biporous HPS (Macronet MN200, Purolite, Llantrisant, UK) via the reduction of [Pd(π-allyl)Cl]_2_ with hydrogen in supercritical CO_2_ has been proposed [93]. The catalyst showed a high activity in the hydrogenation of benzene and was used twelve times without a noticeable decrease in the conversion rate. The use of this catalyst for the hydrogenation of other substrates, like toluene, tetralin, and phenol, is possible. The industrial amine-functionalized crosslinked copolymer MN100 containing Rh nanoclusters showed high catalytic activity in the hydroformylation of olefins in supercritical CO_2_ over six catalytic cycles without loss of conversion rate [94]. Ru-containing nanoparticles of mixed composition containing both oxide and metal components dispersed in HPS matrix (Macronet MN200, Purolite, Llantrisant, UK) were tested in the oxidation of D-glucose to D-gluconic acid [95]. The maximum selectivity for D-gluconic acid was 99.8% with a D-glucose conversion of 99%. The authors explain the high catalytic activity by the presence of pores of various sizes, where macropores facilitate mass transfer, and the presence of small meso/micropores ensures a high stability of the composite by preventing the migration of nanoparticles. Sapunov et al. studied the kinetics of the hydrogenation of D-glucose to D-sorbitol on a similar catalyst {Ru/HPS MN270} and proposed two reaction paths: (1) classical interaction of the substrate D-glucose sorbed on the catalyst surface with hydrogen from the reaction medium and (2) interaction of D-glucose with hydrogen flowing from the surface of the catalyst [96]. Ru-containing HPS-based catalysts modified with magnetite (Fe_3_O_4_) and silica dioxide (SiO_2_) showed high catalytic activity in liquid-phase Fischer–Tropsch synthesis with an yield of liquid C_6_–C_12_ hydrocarbons up to 82% [97]. Modified Ru-Fe_3_O_4_-HPS catalysts with magnetic properties were tested in the deoxygenation reaction of stearic acid in supercritical n-hexane [98]. The catalyst showed a selectivity for C_17+_ of more than 86%, which is significantly higher than similar catalysts based on Si and Ce oxides. The HPS supported catalyst was found to retain its catalytic activity and selectivity for at least 10 consecutive cycles, with the catalyst loss estimated to be less than 0.05 wt.%.

Thus, the application scope of heterogeneous catalytic systems based on HPS is quite large and should increase with time.

## 4. HPS-Based Catalysts for the Synthesis and Transformation of Platform Chemicals

### 4.1. Hydrolytic Hydrogenation of Cellulose to Hexitols

Sugar alcohols, including sorbitol, are included in the list of substances with high added value obtained from biomass [3]. Sorbitol is a product of glucose hydrogenation and an industrial raw material [99,100]. It is used in the food, textile, pharmaceutical, and cosmetology industries for the synthesis of vitamin C, surfactants, resins, glycols, lactic acid, etc. [101,102]. The main industrial method for producing sorbitol is the hydrogenation of glucose, which can be obtained from components of plant biomass, like easily hydrolyzed starch or cellulose [103].

**Figure 2:**
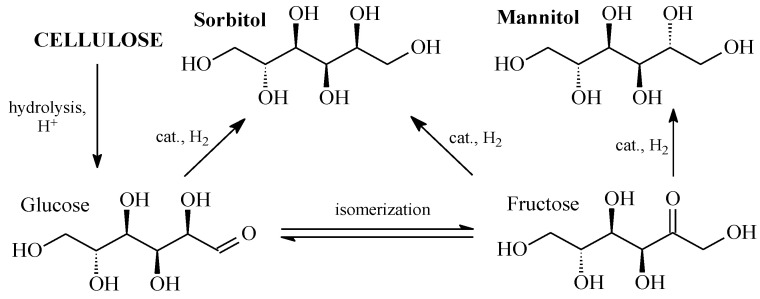
Scheme of hydrolytic hydrogenation of cellulose to hexitols [107].

Cellulose has no nutritional value and comes in large quantities as a waste from the paper, woodworking, and agricultural industries that require disposal. Therefore, using it as a renewable raw material to produce sorbitol is in accordance with the principles of green chemistry, and can be economically beneficial. The presence of many hydroxyl groups in the structure of cellulose determines one of the most effective options for its processing, namely, conversion into hexitols through the hydrolytic hydrogenation [104,105,106], known as one-pot reactions of hydrolysis and hydrogenation (Figure 2). The products of hydrolytic hydrogenation of cellulose are mainly hexitols (sorbitol and mannitol) and some amounts of C_2_–C_5_ polyols formed as a result of hydrogenolysis of monosaccharides and polyols.

The study of simultaneous hydrolysis and hydrogenation of plant polysaccharides began in the 1950s by A.A. Balandin et al., who subjected cellulose to hydrolytic hydrogenation in the presence of mineral acids and Ru-, Pd-, and Pt-containing catalysts [108,109]. For example, in the invention [110] high yield of sorbitol during the hydrolytic hydrogenation of cellulose is claimed in the presence of suspended nickel catalyst at hydrogen pressure of 100–120 atm at 180–200 °C for 40–60 min.

Several decades later, this topic was reconsidered due to the concepts of Green Chemistry [111]. The number of reports on the hydrolytic hydrogenation and hydrogenolysis of cellulose began to increase [112,113]. For environmental reasons, in some cases (under subcritical conditions) the mineral acids as hydrolysis catalysts were replaced by cheap and environmentally friendly water [114,115]. In the late 1990s, Sasaki et al. [116,117] studying the kinetics of dissolution and hydrolysis of cellulose in subcritical and supercritical water showed that in the supercritical region (at a temperature of 374 °C and above) the rate of cellulose hydrolysis is higher than the rate of thermal destruction of glucose, while in subcritical conditions the rate of glucose destruction significantly exceeds the rate of hydrolysis of cellulose macromolecules. Thus, the efficiency of the process of hydrolytic hydrogenation of cellulose carried out in a subcritical water environment will largely be determined by the activity of the catalyst, its ability to quickly and selectively hydrogenate hexoses, which is confirmed by the results of experiments without a catalyst. In the latter case, solutions are formed with an odor and color characteristic of products of the thermal destruction of glucose.

Large numbers of heterogeneous catalytic systems for the direct conversion of cellulose to sorbitol have been proposed. An assessment of the activity of various catalysts in the conversion of biomass into fuel and chemicals showed that catalysts containing noble metals have the highest efficiency [118]. The ruthenium-based catalyst has the maximum activity and comes first in the series: Ru >> Pt ≈ Pd ≈ Au > Rh > Ir >> Os. This is the reason why ruthenium is part of the majority of catalytic systems proposed for the hydrolytic hydrogenation of cellulose and its oligomers with high potential for practical application [119,120,121,122,123,124].

Based on the results obtained in the hydrogenation of mono- and disaccharides with HPS-based catalysts [95,96], our group proposed the use of Ru/HPS catalysts in the hydrolytic hydrogenation of microcrystalline cellulose to produce sorbitol and mannitol [124].

The catalysts were synthesized using the following procedure: HPS was impregnated according to incipient wetness impregnation with the solution of the calculated amount of ruthenium (IV) hydroxochloride in a complex solvent consisting of tetrahydrofuran, methanol, and water at a volume ratio 4:1:1 at room temperature. Then, the catalyst was dried at 70 °C, consecutively treated with solutions of NaOH and H_2_O_2_, and then washed with water until the absence of chloride anions in the washing water. The catalyst was then dried at 85 °C, reduced by hydrogen at 300 °C and atmospheric pressure for 2 h, cooled in nitrogen and kept under air. The catalyst particle size was controlled by sieving (mesh size 60 μm) the initial powdered support. Commercial sorbents HPS MN270 (without functional groups), MN100 (functionalized with amino groups), and MN500 (functionalized with sulfo groups), were purchased from Purolite, UK, and used for the synthesis. The main characteristics of these sorbents and their structure are indicated in Table 1.

The obtained catalysts were tested in the reaction of hydrolytic hydrogenation of cellulose and the results are presented in Table 2.

It was found that the catalyst based on HPS MN270 has the maximum catalytic activity in this process. Its functionalized analogues showed much worse results. Comparing the results of nitrogen physical adsorption (Table 3), one can see that the 1% Ru/MN500 catalyst during the reduction in hydrogen at 300 °C underwent significant structural changes, leading to a more than fivefold decrease in the specific surface area and a huge decrease in the surface of micropores. This is due to a desulfurization of MN500 at temperatures of 100–150 °C [125] affecting the HPS structure.

HPS MN270 and MN100 are thermostable as confirmed by thermogravimetry results [124]. Intensive, multi-stage (probably associated with the rupture of methylene crosslinks) destruction of HPS MN270 begins at about 450 °C. At this temperature the rate of polymer mass loss attains the maximum of 10%/min. A similar behavior is observed for HPS MN100. Intensive destruction of this polymer also begins around 450 °C. However, the maximum rate of mass loss is higher and attains 15%/min. HPS MN100 degrades faster, probably due to the removal of NH_2_ groups. The results obtained suggest that reducing the catalyst based on HPS MN270 and MN100 in hydrogen flow at 300 °C does not affect the HPS morphology.

The main products of hydrolytic hydrogenation of cellulose using a 1% Ru/MN270 catalyst are sorbitol and mannitol; minor products include 1,4-sorbitan, xylitol, erythritol, glycerol, glycols, and some glucose. In addition, trace amounts of glycolic acid, 2-methylpropane-1,2-diol, 3-methylbutane-1,2-diol, butane-1,4-diol, pentane-1,5-diol, hexane-diol, 1,2,6-triol, hexane-1,2,5,6-tetriol, hexane-1,2,3,4,5-pentol, hexane-1,2,3,5,6-pentol, cellobiose, cellotriose, cellotetraose, and other products were found in the reaction medium. The gas phase contains methane and trace amounts of ethane, propane, and isobutane.

Comparing the results obtained with those available in the literature [105,126,127,128,129,130,131,132,133], it should be noted that in the hydrolytic hydrogenation reaction, the 1% Ru/MN270 catalyst showed a fairly high and stable yield of hexitols, a high degree of cellulose conversion, and its use does not require the pretreatment of cellulose (for example, as in most cases, grinding separately or together with a catalyst) or acid addition to accelerate the hydrolysis of cellulose.

### 4.2. Magnetic Catalysts Based on HPS in the Conversion of Plant Polysaccharides

Despite the advantages of homogeneous catalysts, such as high catalytic activity, high selectivity, etc., when creating new industrial catalysts, preference is often given to heterogeneous catalytic systems, which have an important advantage of relatively easy separation from the reaction mixture for subsequent regeneration and reuse [134]. However, existing methods of separation (filtration, centrifugation, decantation, etc.) are laborious, time consuming, and may lead to losses in the catalysts [57], especially if their particles are small in size and density [135]. These problems can be avoided, or at least their impact on production costs can be significantly reduced, if the catalyst particles have magnetic properties [136,137]. Over the past 10–15 years, a large number of magnetic catalysts have been developed and successfully applied for hydrogenation, oxidation, carbon–carbon coupling, click reactions, Suzuki–Miyaura reactions, chiral and enzyme catalysis, photocatalysis [138,139], in transesterification and esterification reactions for the production of biodiesel fuel [140], etc. Magnetic catalyst separation has the following advantages: fast and efficient separation (the process of separating the catalyst by a magnetic field takes seconds or minutes and the catalyst is completely removed); low energy consumption of separation process (by both a permanent magnet and an electromagnet); the catalyst remains inside the reactor and the process can be quickly restarted with minimal catalyst losses after removing the reaction mixture and introducing a new portion of the substrate into the reactor; magnetic catalysts exhibit their properties only in the presence of a magnetic field and there are thus no additional requirements for the storage, handling, and use of such catalytic systems; sampling and product separation are greatly simplified if the reaction takes place in an inert atmosphere; solvent consumption and waste generation are minimized; the process is easily scaled from laboratory to industrial volumes; and magnetic properties can be imparted to any catalysts (based on enzymes, metals, solid acids, etc.) [57]. Magnetic catalysts are currently being actively developed and have great potential for practical applications in industrial catalytic processes [141]. However, they are not yet used on an industrial scale for a number of reasons. First, the synthesis of magnetic metal and metal oxide nanoparticles often requires expensive precursors and toxic organic solvents, which limits large-scale production [139].

Since HPS has proven to be a suitable catalytic support, we have developed a method for the synthesis of magnetic Ru-containing catalysts based on commercial HPS sorbent MN270 (Purolite, UK). The catalyst was prepared using a two-stage procedure. First, magnetite nanoparticles were synthesized in the pores of HPS through the thermal decomposition of iron (III) acetate. At the second stage, Ru-containing nanoclusters were synthesized on the surface of the composite. To achieve this, the composite powder was impregnated with a solution of ruthenium (IV) hydroxochloride in a complex solvent, dried and kept in hydrogen at 300 °C for 2 h. The resulting magnetic catalysts were tested in the conversion of plant polysaccharides, namely in the process of direct conversion of microcrystalline cellulose into glycols: ethylene glycol (EG) and propylene glycol (PG) [142,143]. EG and PG are the most important raw materials for the production of drugs, fuels, surfactants, antifreezes, lubricants and solvents, lactic acid, etc. [144,145]) and in the hydrolytic hydrogenation of inulin to mannitol [146,147].

**Figure 3:**
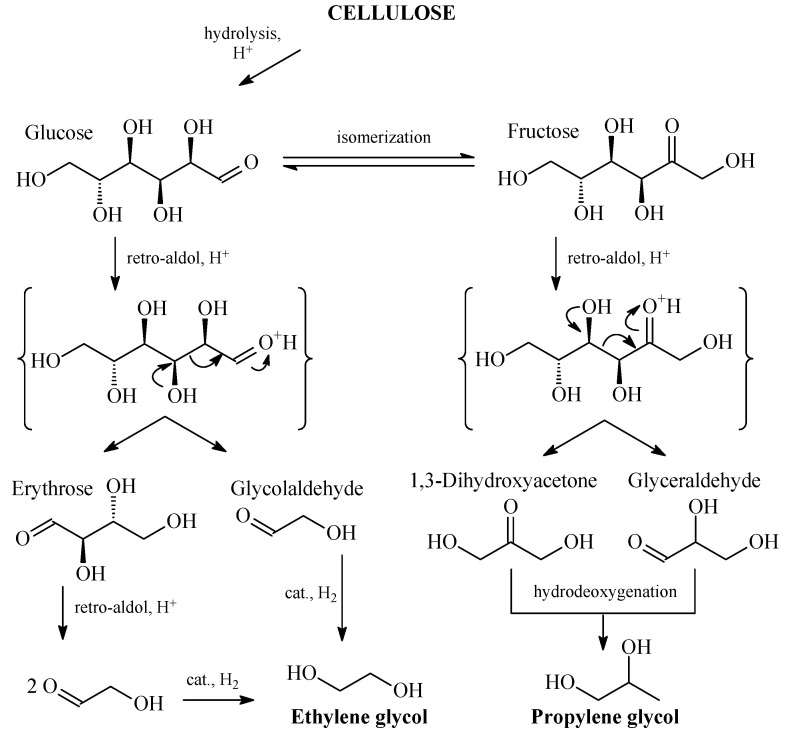
Scheme of the cellulose hydrogenolysis to glycols [107].

The process of conversion of cellulose Into EG and PG is quite complex (Figure 3) and includes several types of reactions, like hydrolysis, isomerization, retro-aldol condensation, hydrogenation, and hydrodeoxygenation [107]. In this regard, development of highly selective catalytic system seems to be rather difficult [148].

The magnetic support (Fe_3_O_4_/MN270) for the Ru-based catalyst was synthesized using the following procedure. FeCl_3_ was dissolved in 95% ethanol. HPS powder (diameter < 45 microns) was put into the solution in the ratio of 1 g HPS per 2 g FeCl_3_, mixed thoroughly and left for 10–15 min. Then sodium acetate was added to this mixture in an amount corresponding to the FeCl_3_/CH_3_COONa ratio of 1/1.5. The mixture was dried until ethanol was completely removed. The resulting red-brown powder was moistened with ethylene glycol, placed in a quartz tube and purged by argon. Then, it was heated up to 300 °C and kept in an argon flow for 5 h. The synthesized Fe_3_O_4_/MN270 powder was washed several times with water, then with ethanol, separated from the solvent with a magnet, and dried to constant weight in an oven at 70 °C in ambient air. The synthesis of 3% Ru-Fe_3_O_4_/MN270 catalyst was carried out using a procedure described in paragraph 4.1 of this review.

Special attention has been paid to the optimization of magnetite particle formation inside HPS pores. Thus, the use of iron (III) nitrate as a precursor [149], turned out to be harmful for the porous structure of HPS (see Table 4). We attributed this result to the formation of strong oxidants during the thermal destruction of nitrate (4Fe(NO_3_)_3_ → 2Fe_2_O_3_ + 12NO_2_ + 3O_2_). The table data show that the specific surface area of such a sample (Entry 3) is about 50-fold smaller as compared to the sample synthesized using FeCl_3_ (Entry 2) as a precursor. It has been shown that the sequential introduction of iron and ruthenium oxides (Entry 4) into the HPS support, the SSA (BET) decreases from 1075 to 364 m^2^/g. Although the proportion of pores with a diameter <6 nm decreases slightly, the samples retain their micro/mesoporous character.

Therefore, co-impregnation by iron (III) chloride and CH_3_COONa was used for the synthesis of Fe_3_O_4_/MN270 composites. The reactions occurring during the synthesis process can be represented as follows:

FeCl_3_ + 3CH_3_COONa = Fe(CH_3_COO)_3_ + 3NaCl.

As a result of the exchange reaction, Fe(CH_3_COO)_3_ is formed in the pores of HPS. It is important to use 95% ethanol as a solvent to prevent hydrolysis of the resulting iron acetate. In the case when hydrolysis does not occur, the subsequent reaction of thermal decomposition of acetate at 300 °C takes place [150]:

6Fe(CH_3_COO)_3_ → 2Fe_3_O_4_ + 9CH_3_COCH_3_ + 9CO_2_ + ½O_2_,

4Fe(CH_3_COO)_3_ → 2Fe_2_O_3_ + 6CH_3_COCH_3_ + 6CO_2_; 2Fe_3_O_4_ + ½O_2_ → 3Fe_2_O_3_.

During these reactions, a significantly smaller amount of oxygen is formed, which is quickly removed by a flow of inert gas, protecting the polymer pore system.

**Figure 4:**
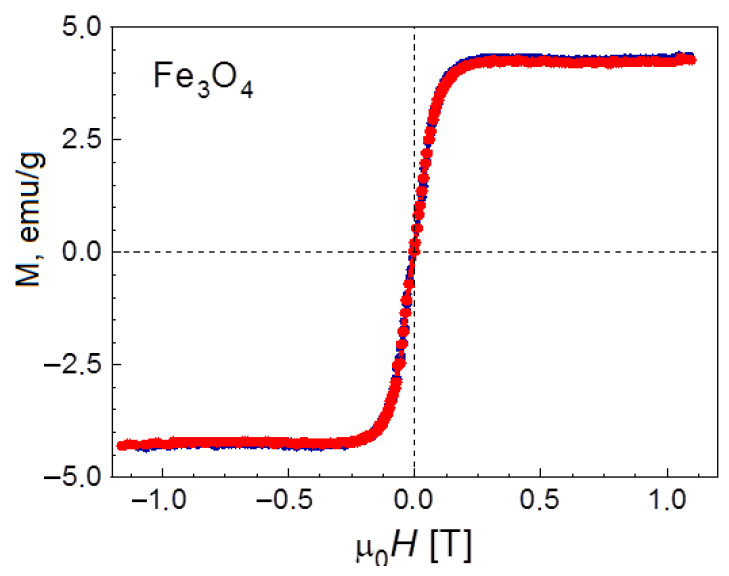
Curves of the Fe_3_O_4_/MN270 sample (red line—magnetization curve; blue line—demagnetization curve) [145].

The synthesized composite was characterized by various methods. First, its magnetic properties were determined and the presence of magnetite nanoparticles with superparamagnetic properties in the pores of HPS was confirmed. Figure 4 shows the magnetization curves of Fe_3_O_4_/MN270 obtained at 25 °C. The absence of hysteresis in the curves indicates the superparamagnetic nature of the material. In addition, a fairly high saturation magnetization value of 4.0 ± 0.5 emu/g was confirmed, which allows the catalyst to be separated from the reaction mixture quickly and fully.

**Figure 5:**
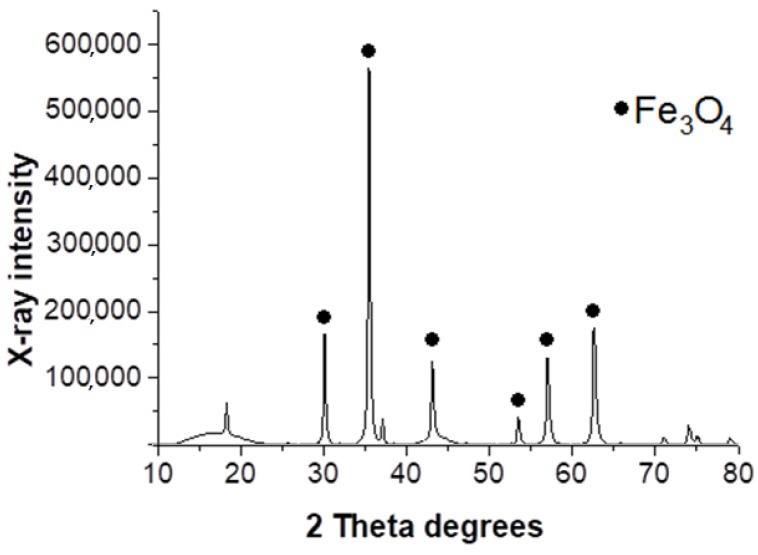
XRD pattern of the Fe_3_O_4_/MN270 [145].

Powder X-ray diffraction data confirmed the magnetite particles formation. The XRD pattern of Fe_3_O_4_/MN270 displays sharp Bragg reflections whose intensity and positions are typical for magnetite (Figure 5).

X-ray fluorescence analysis of the 3% Ru-Fe_3_O_4_/MN270 catalyst gave an Fe content of 19.6 wt.%, and a Ru content of 2.7 wt.%.

Based on TEM results, the mean diameter of Ru nanoparticles of 2.0 ± 0.5 nm and magnetite particles of 40 ± 5 nm was determined.

The synthesized catalyst was used in the hydrogenolysis of microcrystalline cellulose under optimal reaction conditions [149]: 255 °C; 60 bar H_2_; 55 min; 0.3 g of cellulose; 0.07 g of catalyst; 30 mL H_2_O; and 0.195 mol of Ca(OH)_2_ per 1 mol of cellulose. The results obtained are shown in Table 4.

It was found that the glycol selectivity with the 3% Ru-Fe_3_O_4_/MN270 magnetic catalyst is approximately equal to the selectivity of 5% Ru-Fe_3_O_4_-SiO_2_ at the same conversion level [149]. However, due to the lower ruthenium content of the HPS catalyst, its specific activity (calculated per gram of Ru) was higher by 35% for EG and 20% for PG.

A comparison of the results for the 3% Ru/MN270 (Table 5, entry 3) and for the 3% Ru-Fe_3_O_4_/MN270 catalysts shows that Fe_3_O_4_ promotes hydrogenolysis, increasing the yield of glycols. According to [151,152], the activity and selectivity of catalytic hydrogenation are significantly improved when catalytic nanoparticles are supported on iron oxide. Moreover, an intimate contact of catalytic and magnetic nanoparticles can lead to electron transfer from the iron oxide to ruthenium surface [153], facilitating hydrogenation [154].

The reuse of the 3% Ru-Fe_3_O_4_/MN270 catalyst in multiple reaction cycles showed its stability under hydrothermal conditions without any loss of magnetic properties [143]. This is an important advantage for its application in biomass processing, which often is characterized by incomplete conversion of the initial substrates and the formation of large number of byproducts; a separation of the catalyst thus becomes a challenge.

**Figure 6:**
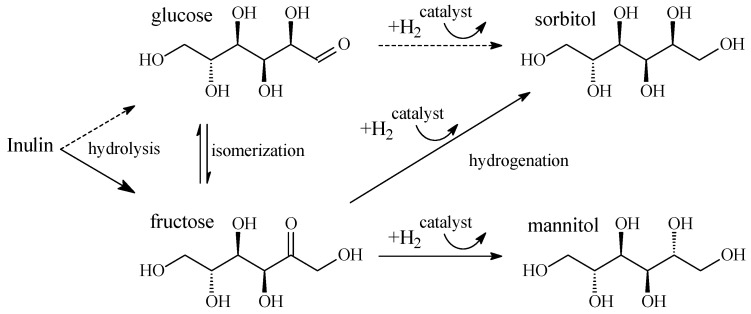
Scheme of the hydrolytic hydrogenation of inulin [107].

This catalyst was also used for the hydrolytic hydrogenation of inulin, a plant polyfructosan (Figure 6). The high content of inulin in some plants allow this polysaccharide to be a promising renewable source for the production of chemicals and fuel [155]. For example, Jerusalem artichoke (*Helianthus tuberosus* L.) contains up to 82% inulin and has great prospects for cultivation. The main product of the reaction is mannitol, which is used, in particular, in the treatment of brain diseases [156], as a food additive, in perfumery, and for the production of varnishes, resins, surfactants, and other products [157,158].

Heinen et al. [159] studied the conversion of inulin to mannitol using Ru-containing activated carbon treated with ammonium persulfate as a catalyst. The maximum selectivity for mannitol was 40%. The authors found a certain amount of short-chain inulin oligomers of the GF_n_ composition (G-glucose, F-fructose) in the solution. The authors explain their presence in the reaction medium after the reaction completion by the fact that the formation of hexitols from inulin occurs simultaneously along two paths: hydrolysis of inulin to monosaccharides with their subsequent hydrogenation and hydrogenation of F_m_ fragments of inulin with their subsequent hydrolysis. The GF_n_ fragments formed during the hydrolysis of inulin do not undergo hydrogenation and accumulate in some quantities in the catalyst. In [160], the process of hydrolytic hydrogenation of inulin was studied in the presence of a Ru-containing homogeneous catalyst based on trisulfonated triphenylphosphine ((TPPTS, P(*m*-C_6_H_4_SO_3_Na)_3_)—Ru-TPPTS. It was shown that after hydrogenation of the main part of fructose, the hydrogenation of glucose, and, consequently, the formation of sorbitol becomes more pronounced, and the mannitol/sorbitol ratio in the final solution decreases. However, with hydrolytic hydrogenation of inulin, the mannitol/sorbitol ratio is approximately 30% higher than with the hydrogenation of a mixture of glucose and fructose. As a result, the authors concluded that the stereoselectivity of the hydrogenation of D-fructose units in partially hydrolyzed inulin oligomers is higher than in the hydrogenation of pure fructose. In [161], Ru-containing catalysts based on a Cs-substituted tungsten phosphate support (Ru/Cs_x_H_3_-xPW_12_O_40_) were proposed for the hydrolytic hydrogenation of cellulose and inulin. The total yield of hexitols (sorbitol and mannitol) during the hydrolytic hydrogenation of inulin was 84%. The authors noted that the new catalyst exhibits high activity, despite the absence of strong internal Brønsted acid sites.

For the first time, magnetic catalysts based on mesoporous silicon dioxide for this process were proposed by our group [162]. Testing a new magnetic catalyst based on HPS in the hydrolytic hydrogenation of inulin to mannitol was carried out under previously determined optimal conditions: 0.1167 mmol Ru per 1 g of inulin; 30 mL H_2_O; 150 °C; and P(H_2_) 60 bar, 45 min. The results obtained are presented in Table 6. Inulin conversion was 100% for both catalysts. It can be seen that the selectivity to mannitol for 3% Ru-Fe_3_O_4_/MN270 is slightly higher than for the 5% Ru-Fe_3_O_4_/SiO_2_ [162]. However, considering the lower ruthenium content in the polymer catalyst, the productivity of 3% Ru-Fe_3_O_4_/SiO_2_ is almost twice as high.

The 3% Ru-Fe_3_O_4_/MN270 was stable when reused in the hydrolytic hydrogenation of inulin. It was shown that the selectivity to mannitol and the catalyst activity do not change after three consecutive reactions, demonstrating excellent stability in the inulin-to-mannitol conversion. Magnetic properties of the catalyst allow easy separation from the reaction mixture without any loss.

In general, the results of our study confirm that magnetic catalytic systems based on HPS are promising in the conversion of plant polysaccharides into substances of high added value.

### 4.3. Hydrolytic Oxidation of Cellobiose to Glucaric Acid

Glucaric acid is an important platform compound used to produce detergents, polymers, and other valuable products [163,164,165]. The glucaric acid market is constantly growing and should reach USD 1.46 billion by 2027 [166]. Currently, glucaric acid is obtained by chemical oxidation of glucose with nitric acid, which is a non-selective, expensive, and environmentally hazardous process [167]. Another option for the synthesis of glucaric acid is oxidation using heterogeneous catalysts through the stage of formation of gluconic acid. The main disadvantage of the existing methods for producing glucaric acid is the use of mono- and disaccharides as raw materials, which have nutritional value (glucose and sucrose). In this regard, plant biomass, which has no nutritional value, is an ideal raw material for the synthesis of acids [168]. Thaore et al. [169] conducted a feasibility study for the production of pure glucaric acid from corn stover by two methods: homogeneous oxidation of glucose with nitric acid and oxidation of glucose with air in the presence of heterogeneous catalysts. The study showed that both options can be economically feasible for industrial use since the costs per 1 kg of product were 2.91 and 2.53 US dollars for homogeneous and heterogeneous oxidation. However, the process using heterogeneous catalysts has about 22% lower environmental impact. In this case, the main problem is the selection of a stable catalyst, which should present a high yield of glucaric acid.

Previously reported results on the HPS-based catalysts for the oxidation of monosaccharides [87,95] suggested that such catalytic systems can be successively applied for producing aldonic and aldaric acids directly from plant biomass. For this purpose, our group synthesized a series of catalysts based on hypercrosslinked polystyrene MN270 containing Pt, Pd, Au, and Ru. The synthesized catalysts were characterized and tested in the conversion of cellobiose to glucaric acid [170].

The synthesis of these catalysts was carried out according to the method given in paragraph 4.1 of this review. The precursors were ruthenium (IV) hydroxochloride, hydrogen hexachloroplatinate (IV) hydrate, sodium tetrachloropalladate (II), and gold (III) chloride hydrate (pure; OJSC Aurat, Moscow, Russia). Thus, using the appropriate precursors, 3% M/MN270 catalysts were synthesized (M = Pt, Pd, Au, Ru).

Table 7 and Table 8 present the results of X-ray fluorescence analysis and of low-temperature nitrogen adsorption used to characterize the synthesized catalysts. The elemental analysis data (Table 7) for metal content had values close to the calculated ones, which indicates the consistency of the method used for the synthesis of HPS-based catalysts. From the data in Table 8, it follows that the samples had predominant microporosity with a highly developed internal surface. After introducing metal nanoclusters into the polymer matrix, a change in its characteristics was observed. The specific surface area decreased due to blockage of micro-, meso- and macropores by nanoparticles of the active phase. At the same time, the microporous nature of all samples was preserved.

**Figure 7:**
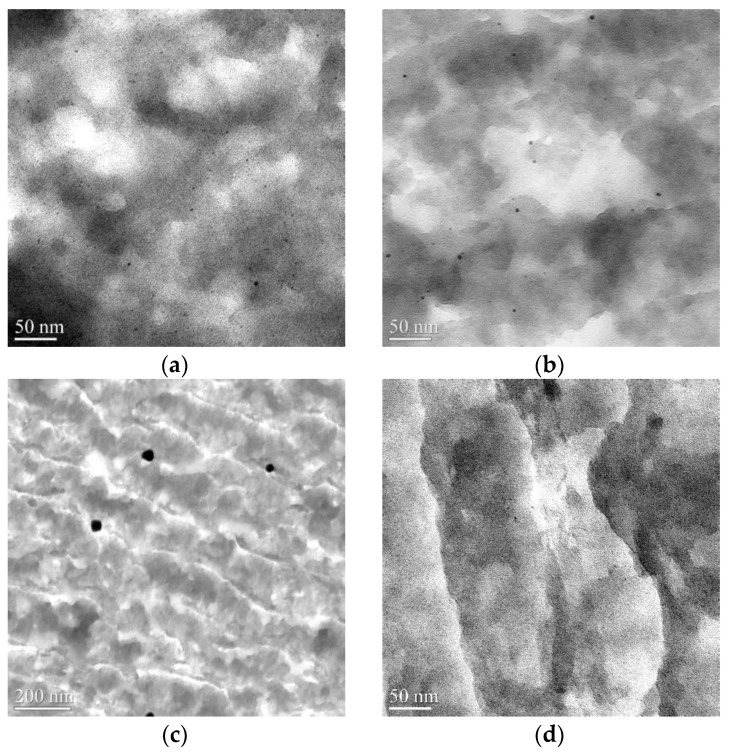
TEM images of catalysts (**a**) 3% Pt/MN270; (**b**) 3% Pd/MN270; (**c**) 3% Au/MN270; (**d**) 3% Ru/MN270 [170].

The synthesized catalysts were characterized via transmission electron microscopy (TEM). The images and the average diameters of metal clusters were obtained (Figure 7). The average size of platinum nanoclusters was 2.8 nm; palladium—3.4 nm; and ruthenium—1.8 nm. The diameter of gold nanoclusters turned out to be approximately an order of magnitude larger, 32.1 nm. It should be noted that nanoparticles of all metals were uniformly distributed within the catalyst volume, and there was no metal crust on the surface of the polymer. We hypothesize that the large size of gold nanoparticles is likely due to the nature of the precursor, interaction with the hydrophobic polymer matrix of the support, and the tendency of gold nanoparticles to aggregate. In our recent work, large gold particles (19.3 ± 8.7 nm) were also obtained using a similar method for the synthesis of the 0.5%-PdAu/HPS-R catalyst [171]. In [172], with a gold content of 1 wt.% in the catalyst, the size of Au-containing nanoparticles was also relatively large (10.9 nm or more). Since our catalyst contains significantly more gold (3 wt.%), the particles formed are larger.

The synthesized catalysts were tested in the hydrolytic hydrogenation of cellobiose. The results obtained are presented in Table 9. The maximum yield of gluconic and glucaric acids was observed with the 3% Pt/MN270 catalyst and attained 16.1% and 41.5% at 100% conversion of cellobiose. Such a high efficiency of the catalyst can be attributed to a much larger number of active centers on its surface as compared to other catalysts [170].

The 3% Au/MN270 catalyst was less effective in the cellobiose oxidation reaction. After the end of the experiment, a fairly large amount of glucose and cellobionic acid, as well as a small amount of gluconic acid, were found in the catalyst. Glucaric acid was present in trace quantities. The conversion of cellobiose was 86.2%. The low activity of the Au-containing catalyst could be due to the large particle size of the active phase. Catalysts containing Pd and Ru showed the worst results: low conversion of the initial substrate and extremely low yields of gluconic and glucaric acids. The experiment without a catalyst showed that the catalyst plays the main role in the cellobiose hydrolysis reaction and that the degree of hydrolysis obviously depends on the nature of the metal in the catalyst.

The process conditions were optimized (temperature of 145 °C, an O_2_ pressure of 5 bar, and a substrate/catalyst mass ratio of 4/1), and the obtained yields of gluconic and glucaric acids reached 21.6 and 63.4%, respectively, at 100% of cellobiose conversion. The maximum yield of gluconic acid was observed after 1 h of reaction while the maximum yield of glucaric acid was after 2 h.

**Figure 8:**
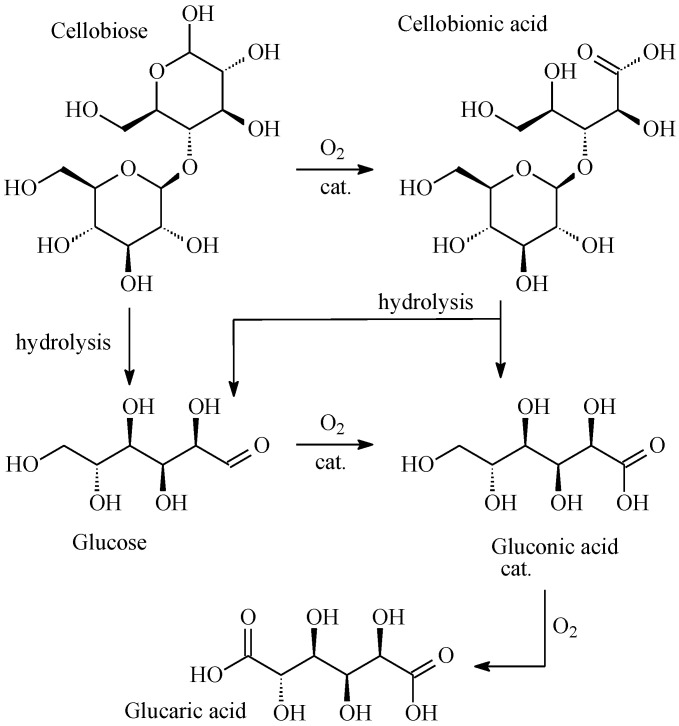
Proposed scheme for the conversion of cellobiose into gluconic and glucaric acids in the presence of the 3% Pt/MN270 catalyst [170].

In the process of the conditions optimization for the hydrolytic oxidation of cellobiose, the obtained qualitative and quantitative results were analyzed and allowed to propose reaction scheme for the conversion of cellobiose into gluconic and glucaric acids in the presence of 3% Pt/MN270 (Figure 8).

The study showed that with quadruple use of 3% Pt/MN270, the yields of gluconic and glucaric acids gradually decreased by 5.5 and 11.0%, respectively, which is likely due to the gradual degradation of the HPS polymer as a result of oxidation, as evidenced by the deterioration of porous properties (Table 10). At the same time, TEM results showed that the average size of Pt particles on the used catalyst remained almost unchanged, at 2.9 nm.

The results obtained are promising for a technology of the catalytic conversion of plant polysaccharides, primarily cellulose, into aldonic and aldaric acids, which are high value products widely used in the chemical, food, pharmaceutical, and other industries.

### 4.4. Hydrogenation of Furfural

**Figure 9:**
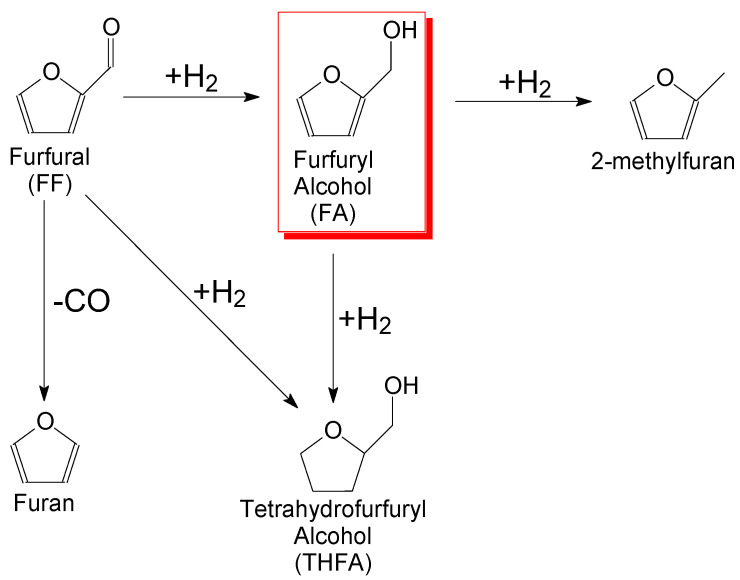
Schematic representation of the FF hydrogenation (the target reaction product is highlighted in red). Reprinted from ref. [175], Copyright 2020, with permission from John Wiley and Sons (Hoboken, New Jersey, USA).

Technological advances in the field of biomass conversion make it possible to produce a wide range of products of different chemical natures, in particular, furfural (FF), which belongs to the furan group. These compounds are widely used in chemical synthesis because they are highly reactive [173,174]. FF can be produced by sequential hydrolysis and dehydration reactions of xylans that are obtained from biomass. Selective hydrogenation of furfural is important reaction in the production of furfuryl alcohol (FA), methylfuran, tetrahydrofurfuryl alcohol, and other compounds (Figure 9).

Furfuryl alcohol is mainly used for the production of special resins, lubricants, plasticizers, polymers and the coatings based on them, etc. [176]. Considering that FF hydrogenation is a complex multi-stage process that occurs with the formation of a large number of products, the synthesis and selection of a suitable catalytic system is very important.

The Pd-containing catalysts based on HPS type MN270 were proposed for the FF hydrogenation. The influence of the precursor nature on the structure, composition, and catalytic properties was studied [177]. Bis(acetonitrile) palladium chloride (PdCl_2_(CH_3_CN)_2_) and palladium acetate (Pd(CH_3_COO)_2_) were used as palladium precursors. All catalysts under study were synthesized via incipient wetness impregnation. The selected Pd (II) precursors have different polarities, which influence their compatibility with HPS and, accordingly, the formation of palladium nanoparticles. The dielectric constants of acetonitrile and acetate are 36.64 and 6.2, respectively [178]. Our previous work [89] showed that HPS is extremely hydrophobic, but due to its unusual porosity and high crosslink density, it can accommodate even completely polar compounds. We believe that the behavior of any compound introduced into a porous matrix depends on its ability to either propagate along the pore walls, when both the matrix and the metal compound have similar properties, or to be repelled from the pore walls (when both are particularly different). This results in particles of different sizes and arrangements depending on the properties of the metal precursor. Therefore, the result depends on the balance of hydrophobicity and hydrophilicity between the metal precursor and HPS [89]. The study using the low-temperature nitrogen physisorption of initial HPS samples and final catalysts showed that the specific surface area of HPS after impregnation with Pd precursors decreased, suggesting pore clogging. It should be noted that the volume of mesopores decreased, while the volume of micropores remained unchanged. These data indicate the formation of palladium nanoparticles in HPS mesopores.

**Figure 10:**
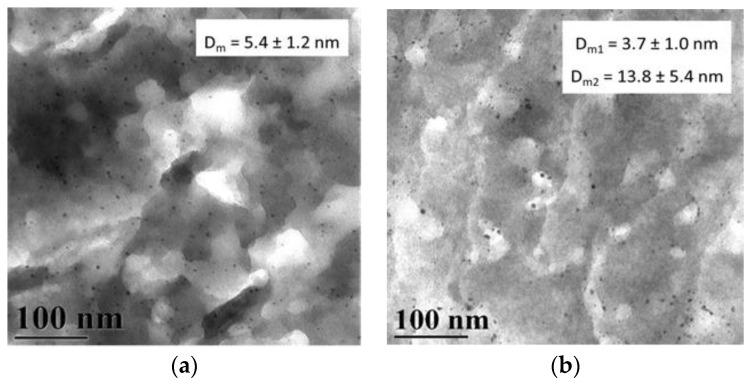
TEM images of the 3% Pd/HPS (PdCl_2_(CH_3_CN)_2_) (**a**) and 3% Pd/HPS (Pd(CH_3_COO)_2_) (**b**). Reprinted from ref. [177], Copyright 2019, with permission from Elsevier (Amsterdam, The Netherlands).

The TEM data (Figure 10) show for the 3% Pd/HPS composite with the PdCl_2_(CH_3_CN)_2_ precursor, a relatively narrow distribution of Pd nanoparticles with an average diameter of 5.4 ± 1.2 nm (Figure 10a). When using the more hydrophobic precursor Pd(CH_3_COO)_2_, a bimodal distribution of particles with average particle sizes of 3.7 ± 1.0 nm and 13.8 ± 5.4 nm is seen (Figure 10b). This observation is consistent with the literature, in that the behavior of metal species in HPS depends on the hydrophobic-hydrophilic balance. The obtained TEM data are also consistent with the BET results, and confirm Pd nanoparticles formation in mesopores rather than micropores.

**Figure 11:**
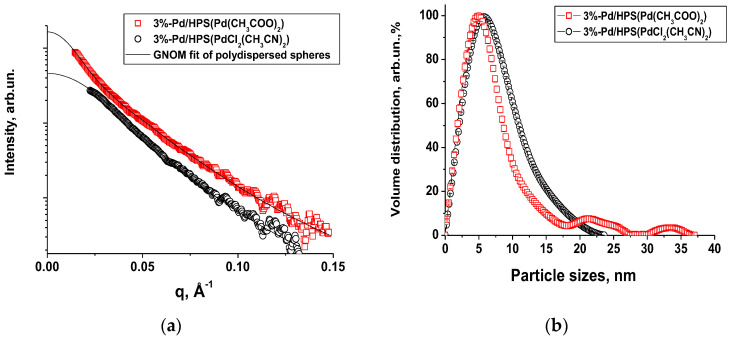
SAXS data for 3% Pd/HPS catalysts after their impregnation with masking liquids (**a**). Volume particle size distributions of Pd particles in the catalysts (**b**). Reprinted from ref. [177], Copyright 2019, with permission from Elsevier (Amsterdam, The Netherlands).

The results obtained from small-angle X-ray scattering (SAXS) showed that in the case of 3% Pd/HPS (PdCl_2_(CH_3_CN)_2_), Pd nanoparticles form a monomodal distribution with an average diameter of 7.5 nm. For the 3% Pd/HPS composite (Pd(CH_3_COO)_2_) (b), there are two fractions: a main fraction of small particles (≈7 nm) and a small amount of larger particles (10–35 nm) (Figure 11). In general, these results are consistent with the TEM data.

**Figure 12:**
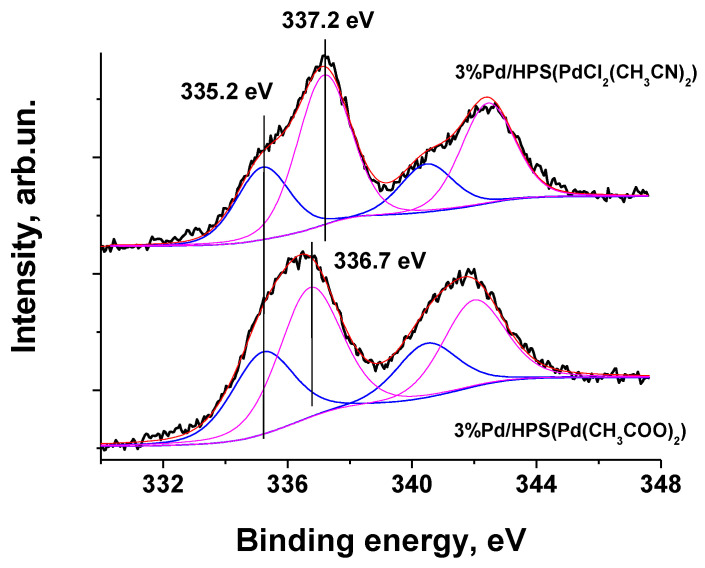
Pd 3d XPS spectra for both Pd/HPS samples and their deconvolution on metal and oxide form of Pd (black line—raw spectrum; red line—synthetic spectrum; blue line—chemical state of palladium Pd^0^; purple line—chemical state of palladium Pd^2+^). Reprinted from ref. [177], Copyright 2019, with permission from Elsevier (Amsterdam, The Netherlands).

XPS data confirm the Pd^0^ nature and oxide form of Pd nanoparticles using both precursors. The XPS spectrum of 3% Pd/HPS(PdCl_2_(CH_3_CN)_2_) is deconvoluted into two components: with Pd 3d_5/2_ binding energies of 335.2 eV and 337.2 eV, which we assign to Pd^0^ (32%) and Pd^2+^ (68%), respectively (Figure 12). In the case of 3% Pd/HPS(Pd(CH_3_COO)_2_) Pd 3d spectrum is also deconvoluted into two components: with Pd 3d_5/2_ binding energies of 335.2 eV (40%) and 336.7 eV (60%) which we also assign to Pd^0^ and Pd^2+^, respectively (Figure 12). The typical values of Pd 3d_5/2_ binding energy for Pd^0^ state is about 335.0–335.4 eV and the range of 336–337 eV corresponds to PdO. As the samples were kept on air before XPS study, some part of metal Pd is present in oxide form. The ratio of oxide form/metal form is higher in more disperse samples because small particles are more easily oxidized compared to larger particles. This value could be used to estimate of the ratio between small and large particles in series of such samples. Also, it could be concluded that the nature of Pd precursor in HPS matrix essentially does not influence the Pd oxidation state.

A comparison of the catalytic activity of Pd-containing catalysts showed that a more dispersed sample (3% Pd/HPS (PdCl_2_(CH_3_CN)_2_) showed higher values of FF conversion and selectivity towards FA due to the presence of smaller particles catalytically active phase. After 200 min of the reaction, FA selectivity for both catalysts reached its maximum–87.4% for 3% Pd/HPS(PdCl_2_(CH_3_CN)_2_) and 83.8% for the 3% Pd/HPS (Pd(CH_3_COO)_2_) catalyst at FF conversion of 55.6% and 36.3%, respectively).

**Figure 13:**
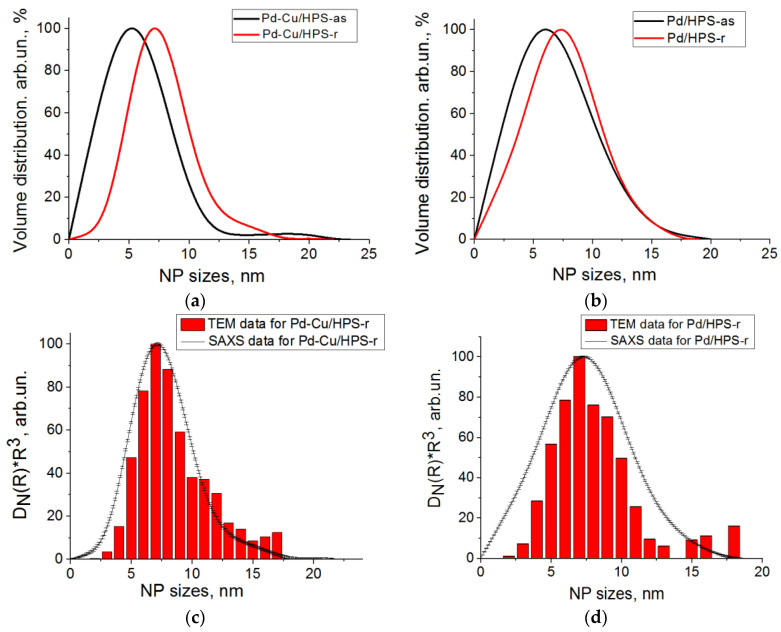
Volume NP size distribution from the SAXS data (**a**,**b**), and a comparison of the NP sizes from TEM and SAXS data (**c**,**d**) for Pd–Cu/HPS-r (**a**,**c**) and Pd/HPS-r (**b**,**d**). Reprinted from ref. [175], Copyright 2020, with permission from John Wiley and Sons (Hoboken, New Jersey, USA).

Considering good catalytic properties of Pd–Cu alloys in the hydrogenation of FF to FA [179] and the advantages of micro/mesoporous HPS supports in a number of hydrogenation reactions [70,124], we developed new catalysts with Pd–Cu alloy nanoparticles in the pores of HPS and compared their properties with those of monometallic Pd nanoparticles [175]. The catalyst was synthesized by impregnating HPS with a solution containing both palladium and copper acetates, followed by treatment with Na_2_CO_3_ to precipitate Pd–Cu mixed oxide nanoparticles in the pores of HPS. These as synthesized samples are denoted “as”. The reduction of Pd and Cu species was performed prior to the catalytic reaction in the hydrogen flow at 275 °C. The reduced samples are denoted “r”. SAXS and transmission electron microscopy (TEM) methods were used to estimate the size of nanoparticles (Figure 13). The average nanoparticle size determined by using both methods was about 6–7 nm.

XRD data for Pd/HPS-r shows typical Pd^0^ reflections for a monometallic sample and a crystallite size of 6 nm, indicating that the Pd nanoparticles are most likely single crystals. For bimetallic Pd–Cu nanoparticles in the Pd–Cu/HPS-r sample, the X-ray diffraction pattern is almost identical, but its reflections are shifted towards large angles, and the peak positions are between the positions characteristic of the Pd and Cu phases. According to data from the literature, this peak location indicates the formation of the structure of a Pd–Cu bimetallic alloy. The absence of reflections from Cu metal, as well as its oxides or hydroxides, once again confirms the formation of the alloy. The size of Pd–Cu nanoparticles remains ~6 nm.

XPS showed the enrichment of the surface of nanoparticles with Cu atoms, as well as the presence of both zero-valent and cationic forms of Pd and Cu, i.e., heterogeneity of nanoparticles. This structure of Pd–Cu alloy nanoparticles immobilized in HPS provides almost 100% conversion and excellent selectivity towards FA (95.2%). These exceptional performances were attributed to the prevention of furan ring adsorption on Pd due to neighboring Cu species and facilitated desorption of FA, resulting in higher selectivity. Controlled adsorption of hydrogen and FF due to the mixed valence states of Pd and Cu species leads to higher conversion. These factors, as well as the remarkable ability to reuse the catalyst in ten successive reactions, make this catalyst promising for industrial applications.

### 4.5. Hydrogenation of Levulinic Acid

**Figure 14:**
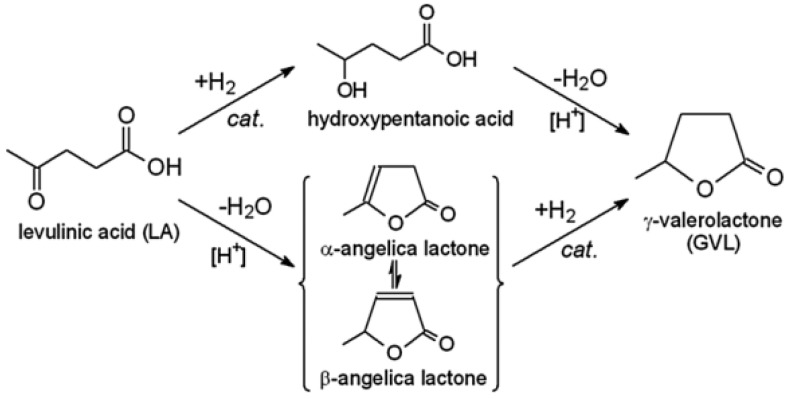
Scheme of LA hydrogenation to GVL. Reprinted from ref. [185], Copyright 2021, with permission from Elsevier (Amsterdam, The Netherlands).

Levulinic acid (LA) is one of the most valuable multifunctional substances obtained from biomass. LA is a precursor to many industrially important chemicals and is widely used in the production of lubricants, chiral reagents, resins, biologically active substances, adsorbents, electronics, and batteries [7,180]. Due to the presence of two highly active functional groups (carbonyl and carboxyl), LA easily enters oxidation, reduction, esterification, substitution, and condensation reactions, which makes it a very valuable platform compound [181]. One of the most important reactions involving LA is hydrogenation to form γ-valerolactone (GVL) (Figure 14), a key reaction in the conversion of plant carbohydrates into renewable fuels and chemicals [182]. GVL can be used as an environmentally friendly solvent, an additive to liquid fuels, and also for the synthesis of polymer precursors such as adipic acid and diols [183,184].

Today, the mostly used in the LA selective hydrogenation to GVL are bifunctional catalysts based on inorganic supports containing Lewis acid sites (LAS) and Brønsted acid sites (BAS). Despite the advantages of bifunctional catalysts in this process, there are some problems caused by the presence of LAS and BAS in the oxide supports. Thus, the high acidity of the support may lead to the formation of coke, which can initiate rapid deactivation of catalysts [186,187,188]. In addition, during the hydrogenation of LA in an aqueous environment, agglomeration and leaching of active metal from the support can occur, which can affect the catalyst stability and significantly complicates its effective reuse [189]. The stability of catalysts can be increased by using carbon supports, which include polymers with a variety of useful properties: high porosity, the presence of functional groups, the ability to vary molecular weight, and hydrophilicity. Balla et al. [190] synthesized uniformly distributed Ni nanoparticles (NPs) with narrow size distribution around 6 nm embedded in a mesoporous carbon substrate (Ni@C) obtained from an organic copolymer. The mesoporous carbon support provided various defect sites for the attachment of Ni particles, and strong interactions between the carbon phase were observed. A total of 100% conversion of LA was achieved in 4 h at 200 °C and 3 MPa in 1,4-dioxane. Balla et al. [191] also synthesized copper NPs (5.5 nm in diameter) embedded in an ordered mesoporous carbon (OMC) carrier by a multicomponent assembly method using chelates. The OMC surface was functionalized with various oxygen-containing functional groups, which enhanced the interaction with copper NPs. The synthesized Cu/OMC catalyst demonstrated high activity and stability in the hydrogenation of LA to GVL in continuous mode (260 °C, 0.1 MPa H_2_) due to the effects of Cu retention in mesoporous carbon. Sychev et al. [192] synthesized catalysts based on ruthenium NPs (with a diameter of about 1.5 nm) deposited on the graphite-like mesoporous carbon material Sibunit-4 (initial and oxidized at different temperatures). The presence of oxygen-containing functional groups on the surface of the support was responsible for the distribution of Ru NPs and the acidic properties of the catalyst. The resulting catalysts containing 1% and 3% (wt.) Ru showed high activity in the hydrogenation of LA to GVL (GVL yield 98 mol.% at 160 °C, 1.2 MPa H_2_).

Common catalytic supports are carbon nanotubes (CNTs) and graphene (Gs), including oxidized ones (GOs) [193]. Zhang et al. [194] used CNTs to support porphyrin (PP) complexes containing ruthenium. Ru-PP/RGO catalyst was prepared in a similar manner. Ru-PP/CNTs and Ru-PP/RGO were used for the hydrogenation of LA and its esters giving GVL. Under optimal conditions (100 °C, 3 MPa), a GVL yield of more than 99% was achieved in 10 h. Sosa et al. [195] synthesized nickel catalysts (10 wt.% Ni) based on CNTs, which showed high activity in the hydrogenation of LA in a continuous mode in a trickle bed reactor at 180 °C and 30 bar. The influence of the nature of nickel precursor (acetate, nitrate, and hydroxide) and the arrangement of nickel particles in CNTs was studied and shown that, depending on the catalyst synthesis conditions, nickel crystallites with diameters from 4 nm to 16 nm were formed. Recently, Wang X. et al. [196] reported a bimetallic Ni/Ru (Ni:Ru = 10:1) catalyst based on ordered mesoporous carbon (Ni/Ru@WOMC). The catalyst was obtained by the self-assembly method using organo-solve lignin as a carbon precursor and Ni^2+^ as a crosslinking agent followed by the addition of Ru. A close to 100% yield of GVL in 2-propanol was attained in 4 h of reaction at 80 °C.

In recent years, there has been a trend to create carbon supports doped by heteroatoms, in particular by nitrogen, for the catalytic transformation of LA [197,198,199,200,201]. For example, Chauhan et al. [198] carried out the hydrogenation of LA using formic acid as a source of hydrogen. The catalyst, Ru-decorated and N-doped carbon nanoplates, provided 99.8% LA conversion and 100% GVL selectivity. Wang D. [199] developed N-doped hierarchical carbons with incorporated Co particles (Co@NC). The Co@NC-700 catalyst (carbonated at 700 °C) provided 100% LA conversion at 190 °C and 1.9 MPa H_2_ for 2 h. Also, the catalyst showed high stability (up to five reuses), which was likely due to the synergy of the metal active sites Co, Co-Ox, and Co-Nx. Li et al. [200] developed catalysts containing ultrafine Ru NPs stabilized in hierarchically porous N-doped carbon nanospheres (HPNCs) obtained by nano-emulsion self-assembly. The Ru/HPNC catalyst demonstrated excellent catalytic performance in the hydrogenation of LA to GVL under solvent-free conditions: GVL yield > 99% in 2 h. Yang et al. [201] synthesized Ru NPs stabilized in three-dimensional hierarchical carbon nanoflowers containing pyridinic nitrogen (Ru/PNC). The role of pyridine N compounds was attributed to the formation of electron-rich Ru, which provided weaker adsorption of LA but stronger adsorption of H_2_ on Ru. This resulted in high activity (TOF 5042.5 h^−1^) and selectivity to GVL > 99%. In addition, a direct correlation was found between TOFs and surface pyridine-N/Ru^0^ ratios.

There are also reports of polymer carriers (dendrimers, polystyrenes, metal−organic frameworks (MOFs), porous organic polymers (POPs), etc.) being used for LA hydrogenation. Polymers are used both for the immobilization of catalytically active complexes [202] and metal-containing (for example, Co, Cu, Pt, Pd, and Ru) nanoparticles [203,204,205,206,207,208,209]. It is important that natural polymers can be used as active phase carriers to create catalysts for the hydrogenation of LA. Thus, in the work of Xu et al. [208] the self-assembly method was applied to synthesize colloidal nanospheres based on lignin containing Co^2+^ ions. In this case, heavy metal ions acted as crosslinking agents. After calcination at 500 °C, particles of CoO and metallic Co were formed, providing a 99.8% yield of GVL with 100% conversion of LA in 60 min at 200 °C and 2 MPa H_2_.

Among polymer carriers, it is worth highlighting polymers containing functional groups that play the role of acid sites (-SO_3_H). However, reports on such polymers in LA transformation are scarce [203,209,210]. For example, Yao et al. [203] developed a bifunctional catalyst based on Ru NPs (diameter about 3 nm) immobilized in crosslinked sulfonated polyether sulfone for the selective hydrogenation of LA to GVL. The combination of acid and metal centers lead to high activity. Interestingly, the LA conversion, achieved in 2 h at a temperature of 70 °C and a pressure of 3.0 MPa, increased from 87.9% to 92.1% after the first hydrogenation and gradually to 97.2% in the fourth cycle suggesting in situ catalyst activation.

Despite a variety of LA hydrogenation catalysts [211], supported ruthenium NPs remain one of the most common catalytic systems. We have previously shown that HPS can be successfully used as a support for the creation of Ru [212,213] and Ru−Co [185] catalysts for the hydrogenation of LA to GVL in an aqueous environment. It was shown that the HPS support allows to synthesize Ru-containing NPs, predominantly consisting of RuO_2_ and exhibiting high activity and selectivity in the LA hydrogenation. At the same time, HPS functionalized with tertiary amino groups (HPS-NR_2_) gives tiny Ru-containing NPs (with a diameter of 1–2 nm), which ensures 100% yield of GVL at a temperature of 100 °C and a partial hydrogen pressure of 2.0 MPa for 100 min of reaction at a LA-catalyst ratio of 100 g/g. The activity of the catalyst based on non-functionalized HPS (5%-Ru/HPS), containing NPs with a diameter of about 4 nm, is inferior to the activity of the 5%-Ru/HPS-NR_2_ sample: the conversion of LC in the case of 5%-Ru/HPS is 83% in 100 min reactions. For the 3%-Ru/HPS-NR_2_ catalyst, GVL yields of 77% and 99% can be achieved under similar conditions at LA-catalyst ratios of 100 g/g and 50 g/g, respectively. It is interesting to note that in non-functionalized HPS, Ru-containing NPs tended to form cluster-like aggregates located closer to the outer surface of the polymer granules, to a greater extent than in the SPH-NR_2_, which is due to different hydrophobicity of the polymers [212]. In addition, during the study of the kinetic of LA hydrogenation in the presence of the 5%-Ru/HPS-NR_2_ catalyst, it was found that the apparent activation energy is about 28 kJ/mol, and it was assumed that the LA hydrogenation in an autoclave-type reactor can be partially limited by the mass transfer of hydrogen from the gas phase to the aqueous solution [213].

In a study of bimetallic samples, it was shown that the most promising modifier metal for ruthenium is cobalt. It was found [183] that bimetallic Ru−Co catalysts based on HPS-NR_2_ containing 3% (mass) Ru provide 99% yield of GVL at 120 °C and a partial hydrogen pressure of 2 MPa for 60 min of reaction. Compared to the monometallic analogue (3%-Ru/HPS-NR_2_), the most promising bimetallic catalytic system (3%-Ru-0.1%-Co/HPS-NR_2_) increases the initial reaction rate by approximately 1.5 fold, probably due to the redistribution of RuO_2_ NPs inside the polymer after its impregnation with a cobalt precursor. It is noteworthy that no chemical reaction products of Co and Ru were detected on the catalyst surface, which could be a consequence of the low cobalt amount. A study of the stability of the 3%-Ru-0.1%-Co/HPS-NR_2_ catalyst showed only slight decrease in activity: the LA conversion was 82% after three cycles. The results are very promising for an industrial application.

## 5. Conclusions

Hypercrosslinked porous materials, in particular hypercrosslinked polystyrene, HPS, are already widely used in a variety of industrial processes, mainly as excellent adsorbents. Considerable knowledge has been also accumulated on their application as support for heterogeneous catalysts, including the processes of biomass processing and transformations of platform compounds. Platform chemicals, also known as chemical building blocks, are substances that serve as starting materials for the synthesis of various value-added products. The volume of platform chemicals derived from biomass is constantly growing due to the demand of sustainability for chemical industry.

This review focused on the recent advances in this field, giving several examples of the use of catalysts based on nanoparticles of catalytically active metals stabilized by a polymer matrix of HPS. The main advantages of HPS-based catalysts could be summarized as following: (1) large specific surface area with macro-, meso-, and microporosity; (2) chemical and thermal stability; (3) the possibility of functionalization for controlling surface hydrophobicity and interaction with metal precursors; (4) the possibility of synthesizing catalytic composites with magnetic properties for easy separation and reuse; (5) the ability to control morphology of active nano-particles; and (6) low cost of the HPS supports, which increases economic feasibility of the processes under development.

At the same time, some problems are associated with the HPS as catalytic support. Drawbacks include: (1) difficult access of big reagent molecules into the small pores of the catalyst; (2) the absence of surface acid sites, which are important for some reactions, like hydrolysis; (3) a special attention is required to the nature of the active phase precursor (due to its interaction with the hydrophobic polymer); and (4) the limited temperature regime (below 300 °C), which could be applied during catalyst synthesis and chemical reactions. These challenges need to be overcome to extend the industrial applications of HPS-based catalysts for novel processes of biomass transformations into platform chemicals.

## Figures and Tables

**Table 1:** Main characteristics of MN type sorbents.

Specification	HPS		
	MN270	MN100	MN500
Structure of the polymer matrix	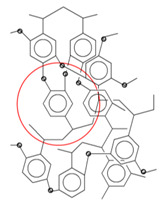	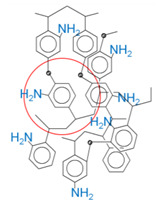	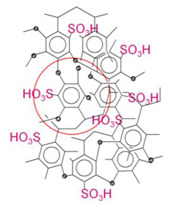
Functional groups	-	Tertiary amine	Sulfonic acid
Specific surface area	~1400 m^2^/g	~900 m^2^/g	~600 m^2^/g
Character of porosity	micro-mesoporous with a predominance of micropores	micro-mesoporous with a predominance of micropores	micro-mesoporous with a predominance of micropores
Pore volume	~0.4 mL/g	~0.4 mL/g	~0.3 mL/g

**Table 2:** Dependences of the cellulose conversion, total yield of hexitols (***η_hex_****.*) and selectivity to sorbitol (S) and mannitol (M).

Catalyst	Cellulose Conversion, %	*η_hex._*, %	Selectivity, %	
			S	M
1.0% Ru/MN270	84.3	50.4	50.5	9.3
1.0% Ru/MN100	77.0	12.7	14.9	1.6
1.0% Ru/MN500	80.6	1.8	1.8	0.4
Without a catalyst	55.6	0.5	0.6	-

245 °C, 6 MPa H_2_, 30 mL water, 600 rpm, 0.028 mmol Ru on 1 g cellulose, process duration 5 min.

**Table 3:** Porosity data for the HPS samples and the catalysts.

Sample	Surface Area		
	Langmuir	BET	t-Plot
	S_L_, ^a^ m^2^/g	S_BET_, ^b^ m^2^/g	S_t_ ^c^, m^2^/g
MN270	1500	1420	295 ^d^ 1140 ^e^
MN100	890	740	195 ^d^ 600 ^e^
MN500	650	540	150 ^d^ 450 ^e^
1% Ru/MN270	1270	1180	250 ^d^ 990 ^e^
1% Ru/MN100	840	730	200 ^d^ 590 ^e^
1% Ru/MN500	120	90	80 ^d^ 15 ^e^

^a^ S_L_ is the specific surface area (Langmuir model); ^b^ S_BET_ is the specific surface area (BET model); ^c^ S_t_ is the specific surface area (t-plot); ^d^ specific surface area according to a t-plot model; ^e^ specific surface area of micropores.

**Table 4:** Porosity data for the HPS and the Fe_3_O_4_/MN270 samples.

Entry	Sample	S_L_, ^a^ m^2^/g	S_BET_, ^b^ m^2^/g	S_t_ ^c^, m^2^/g
1	HPS MN270	1075	1191	265 ^d^; 807 ^e^
2	Fe_3_O_4_/MN270 (FeCl_3_)	450	480	160 ^d^; 289 ^e^
3	Fe_3_O_4_/MN270 (Fe(NO_3_)_3_)	11	9	30 ^d^; 0 ^e^
4	3% Ru Fe_3_O_4_/MN270	364	392	175 ^d^; 189 ^e^

^a^ S_L_ is the specific surface area (Langmuir model); ^b^ S_BET_ is the specific surface area (BET model); ^c^ S_t_ is the specific surface area (t-plot); ^d^ specific surface area according to a t-plot model; ^e^ specific surface area of micropores.

**Table 5:** Catalytic activity/selectivity in the hydrogenolysis of microcrystalline cellulose.

Catalyst	Cellulose Conversion, %	Selectivity, %		Specific Catalytic Activity in Gram of EG (or PG) g^−1^ Ru h^−1^	
		EG	PG	EG	PG
3% Ru-Fe_3_O_4_/MN270	100	22.6	20.0	39.12	34.62
5% Ru-Fe_3_O_4_-SiO_2_	100	19.1	20.9	25.29	27.72
3% Ru/MN270	95	7.2	12.3	7.51	12.71

**Table 6:** Selectivities (S_m_) and catalytic activities for mannitol production.

Catalyst	S_m_, %	Specific Catalytic Activity Calculated as Mass of Mannitol/Mass of Catalyst·h^−1^	Specific Catalytic Activity Calculated as Mass of Mannitol/Mass of Ru·h^−1^
5% Ru-Fe_3_O_4_/SiO_2_	44.3	2.53	50.67
3% Ru-Fe_3_O_4_/MN270	48.7	2.78	92.76

**Table 7:** Results of X-ray fluorescence analyses of the synthesized catalysts.

Catalyst	M Loading, wt.%	M Content from Elemental Analysis, wt.%
3% Pt/MN270	3.00	2.91
3% Pd/MN270	3.00	2.95
3% Au/MN270	3.00	2.87
3% Ru/MN270	3.00	2.70

**Table 8:** Results of the studies of the initial sample of HPS and the catalysts via the low-temperature nitrogen adsorption.

Sample	BET	Langmuir	t-Plot	
	S_BET_, m^2^/g	S_L_, m^2^/g	S_t_, m^2^/g	V, cm^3^/g
HPS MN270	1075	1191	265 ^1^, 807 ^2^, 1072 ^3^	0.37
3% Pt/MN270	863	944	184 ^1^, 678 ^2^, 862 ^3^	0.31
3% Pd/MN270	649	758	94 ^1^, 553 ^2^, 647 ^3^	0.22
3% Au/MN270	738	810	141 ^1^, 593 ^2^, 734 ^3^	0.25
3% Ru/MN270	839	921	151 ^1^, 699 ^2^, 856 ^3^	0.28

^1^ Specific surface area surface of meso- and macropores. ^2^ Specific surface area of micropores. ^3^ Total specific surface area. S_L_—specific surface area (Langmuir model); S_BET_—specific surface area (BET model); S_t_—specific surface area (t-plot); V—volume of micropores.

**Table 9:** Cellobiose conversion and selectivity to the main reaction products for the catalyst with different active phase.

Catalyst	Cellobiose Conversion, %	Product Selectivity, %				
		Glucose	Cellobionic Acid	Gluconic Acid	Glucaric Acid	Σ of Byproducts
3% Pt/MN270	100	4.1	9.4	16.1	41.5	28.9
3% Au/MN270	86.2	24.6	50.1	12.3	0	13.0
3% Pd/MN270	53.3	24.2	40.0	2.8	0	33
3% Ru/MN270	45.4	26.4	0	0	0	73.6
blank (without catalyst)	9.5	14.7	0	0	0	85.3

Cellobiose, 0.2 g; catalyst, 0.05 g; H_2_O, 20 mL; 145 °C; O_2_, 5 bar; 1 h. Byproducts: acetic acid, succinic acid, oxalic acid, glycolic acid, glyceric acid, formic acid [173], and products of monosaccharide caramelization.

**Table 10:** The characterization of the initial catalyst and the catalyst after four reaction cycles.

Sample	BET	Langmuir	t-Plot	
	S_BET_, m^2^/g	S_L_, m^2^/g	S_t_, m^2^/g	V, cm^3^/g
3% Pt/MN270 (initial)	863	944	184 ^1^, 678 ^2^, 862 ^3^	0.31
3% Pt/MN270 (used)	324	345	99 ^1^, 224 ^2^, 323 ^3^	0.10

^1^—specific surface area surface of meso- and macropores; ^2^—specific surface area of micropores; ^3^—is the total specific surface area; S_L_—specific surface area (Langmuir model); S_BET_—specific surface area (BET model); S_t_—specific surface area (t-plot); V is the volume of micropores.
